# Multi-level analyses of spatial and temporal determinants for dengue infection

**DOI:** 10.1186/1476-072X-5-5

**Published:** 2006-01-18

**Authors:** Sophie O Vanwambeke, Birgit HB van Benthem, Nardlada Khantikul, Chantal Burghoorn-Maas, Kamolwan Panart, Linda Oskam, Eric F Lambin, Pradya Somboon

**Affiliations:** 1 Department of Geography, Université catholique de Louvain, Place Pasteur, 3, 1348 Louvain-la-Neuve, Belgium; 2 KIT (Koninklijk Insituut voor de Tropen/Royal Tropical Institute), KIT Biomedical Research, Amsterdam, Meibergdreef 39, 1105 AZ Amsterdam, The Netherlands; 3 Office of Vector Borne Disease Control No.2, 18 Boonruangrit Road, Muang District, Chiang Mai 50200, Thailand; 4 Institute of Virology, Erasmus University Rotterdam, the Netherlands; 5 Department of Parasitology, Faculty of Medicine, Chiang Mai University, Chiang Mai 50200, Thailand

## Abstract

**Background:**

Dengue is a mosquito-borne viral infection that is now endemic in most tropical countries. In Thailand, dengue fever/dengue hemorrhagic fever is a leading cause of hospitalization and death among children. A longitudinal study among 1750 people in two rural and one urban sites in northern Thailand from 2001 to 2003 studied spatial and temporal determinants for recent dengue infection at three levels (time, individual and household).

**Methods:**

Determinants for dengue infection were measured by questionnaire, land-cover maps and GIS. IgM antibodies against dengue were detected by ELISA. Three-level multi-level analysis was used to study the risk determinants of recent dengue infection.

**Results:**

Rates of recent dengue infection varied substantially in time from 4 to 30%, peaking in 2002. Determinants for recent dengue infection differed per site. Spatial clustering was observed, demonstrating variation in local infection patterns. Most of the variation in recent dengue infection was explained at the time-period level. Location of a person and the environment around the house (including irrigated fields and orchards) were important determinants for recent dengue infection.

**Conclusion:**

We showed the focal nature of asymptomatic dengue infections. The great variation of determinants for recent dengue infection in space and time should be taken into account when designing local dengue control programs.

## Introduction

Dengue is a mosquito-borne viral infection, now endemic in most tropical countries, and a major public health concern [1]. The reasons for the global resurgence of epidemic dengue fever are not fully understood but are related to demographic and societal changes, including increased population movements. In Thailand, dengue fever/dengue hemorrhagic fever has been classified a leading cause of hospitalization and death among children [1]. Epidemics of dengue have been reported throughout the country, with large outbreaks in 1987 and 1998 [2,3], and are spreading from Bangkok [4].

Dengue virus is transmitted by *Aedes *mosquitoes. The container-breeding *Aedes *(*Stegomyia*) *aegypti *(=*Stegomyia aegypti *of [6], see below) became important following rapid urbanization in the 20^th ^century [5]. *Aedes *(*Stegomyia*) *albopictus *(=*St. albopictus *of [6]), although having a controversial role in dengue transmission [7], is found in artificial and natural containers in rural and peri-urban areas [5]. Sparse vegetation, low altitude and good transportation routes favor *Ae. aegypti *over *Ae. albopictus *[8]. As a result of phylogenetic studies of the mosquito tribe Aedini, Reinert *et al*. [6] proposed generic status for a number of traditionally recognized subgenera of genus *Aedes*, including *Stegomyia*. However, the traditional classification of genus *Aedes*, with *Stegomyia *as a subgenus, is used in this paper.

Many factors have been associated with dengue transmission, including urbanization, water storage and inadequate water supply, increase in discarded containers, and population movements [9]. Marked spatial and temporal diversity in dengue incidence indicated the complexity of dengue virus transmission in a school population in Thailand [10].

Changes in dengue incidence over time and space might also be caused by changes in land use. In Thailand, great areas of forest have been cleared for cash crops and orchards; rice fields have been converted into housing in peri-urban areas, potentially increasing the area and conditions suitable for vector breeding. Only a few attempts have been made at linking land cover or spatial features to dengue infection since it was generally accepted that dengue transmission was restricted to urban areas and settlements rather than natural or agricultural environments [11,12]. However, prevalence of seropositivity was recently found to be equally high in rural and peri-urban sites, but risk factors have been shown to vary between rural areas and between rural and peri-urban sites [13]. Analyzing land cover and land use is also relevant because of the links between mosquito breeding and land cover [Vanwambeke et al., forthcoming], and people's location in relation to these.

Since passive surveillance is used in Thailand, many infections are missed because of this large proportion of asymptomatic infections. In a prospective cohort study in Thailand, 87% of the dengue virus infections were sub-clinical [14]. Seropositivity can be used as a marker for dengue infection.

The present study was undertaken to investigate personal, household, and environmental determinants for recent dengue infection and how these vary over space and time. A Geographical Information System (GIS) was used to evaluate determinants related to landscape features such as land cover.

## Methods

### Study design

**Figure 1:**
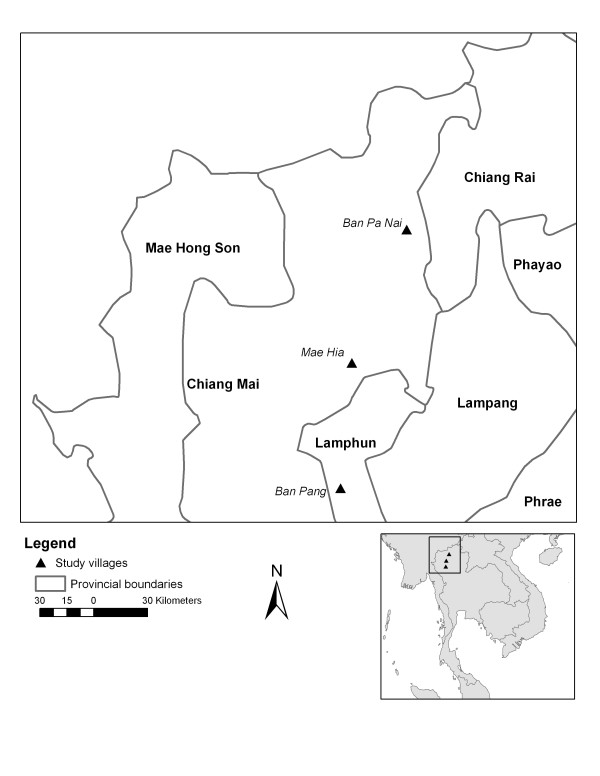
Location of study sites.

Three study sites with changes in land cover between 1989 and 2000 were selected based on patterns of change observed on Landsat images and field visits (Figure 1).

• Ban Pa Nai is a rural area in Chiang Mai province with two villages at an altitude of 450 m. The main land use change observed is the intensification of irrigated areas, facilitated by a dam built in 1996 and by the use of dry season crops.

• Ban Pang is a rural site in Lamphun province at an altitude of 380 m. Surrounding a narrow irrigated valley, large areas on the hill-slope have been cleared for planting longan trees (fruit cash crop).

• Mae Hia is situated in the suburbs of Chiang Mai and is composed of two villages at an altitude of 320 m. Following land speculation and development, large areas of former rice fields were converted into housing projects or left unused following the Asian financial crisis of 1997.

All study villages had a history of dengue infection recorded by the local public health authorities.

Land cover maps were derived from a March 2000 Landsat image with a spatial resolution of 30 meters using the maximum likelihood classification method, with a global accuracy ranging from 81% in the heterogeneous peri-urban Mae Hia to 86% in Ban Pang and 87% in Ban Pa Nai. Details on image pre-processing and classification of the image are provided in [15].

Details of the study methodology were described elsewhere [16]. Briefly, the Medical Ethical Committee of Chiang Mai University approved the study, and local permission and collaboration were obtained. Surveys were conducted in May and September of 2001–2003. The latitude and longitude of main points in the village, including street corners, were registered using a hand-held GPS (global positioning system; Garmin GPS II). Households were located on the map along these geo-referenced streets using preliminary hand-drawn maps. The village maps result from a combination of several sources of information and were cross-checked with the topographic map. All spatial data (household maps, land cover maps) were set to the projection of the 1/50000 topographic map of Thailand (Royal Thai Survey Department).

All inhabitants were asked to voluntarily participate in the study. Written informed consent was obtained. Each year between the May and September surveys, participants filled in a calendar registering daily where they spent most of that day. People reported fever subjectively. Finger prick blood was collected on filter paper during the May and September surveys (903 TM Paper, Schleicher&Schuell, Den Bosch, the Netherlands) and air-dried in the shade. Within one or two days, all filter papers were stored in a refrigerator (4°C) until antibody detection. After reconstitution of the filter papers in phosphate-buffered saline, antibodies were detected using an anti-dengue IgM capture enzyme linked immunosorbent assay (ELISA) (Focus Technologies, Cypress, CA) [17]. According to the manufacturer of the test, a ratio >1.0 is positive; however, many samples had ratios between 1.0 and 1.3, which were considered to be either aspecific reactions or older dengue infections. A ratio of 1.3 or more compared to the reference was therefore considered as seropositive. In primary dengue infections, the level of IgM antibodies rises shortly after infection and they disappear in most patients two to three months afterwards [18], making them appropriate for studying dengue infection on a seasonal basis. Knowing the relative level of IgG and IgM antibodies is however necessary to distinguish between primary or secondary dengue infections. IgG antibodies can remain present for life but, in a secondary infection, IgM will be relatively more important [19]. Measuring IgM therefore only allows measuring recent dengue infections. A case of dengue infection was defined as a person being IgM-positive after being IgM-negative in the previous survey. Non-cases were defined as persons who remained positive or negative or changed from positive to negative in two consecutive surveys.

Potential individual risk factors were assessed by questionnaire and included demographic factors, knowledge of dengue, location and movements during the day and evening, housing condition factors and use of preventive measures. The same survey methods and questionnaire were used in each site.

### Data analysis

The data presented a nested structure, where individuals are nested within households. Individuals living in the same household share some characteristics, like housing and house surroundings, and are therefore more likely to resemble each other than individuals living in different households. This violates the assumption of independence of observation and can result in spurious significant effects [20]. Moreover, factors at the contextual and environmental level in which the individual is embedded (i.e. household factors) can be significant determinants [21]. Multilevel regression methods were thus chosen for the analysis. These methods allow consideration of within-group (household) and between-group relations and integration of household and individual-level variables [22]. Since our dependent variable is binary (individual were infected or not), we used the logistic form of the multilevel model. The surveys provided measurements at successive time points for each individual, adding a third level to the data [23], where surveys are nested within individuals. The intraclass correlation (ICC) allows calculation, on an empty model, of the proportion of the total variance explained by the grouping structure, in our case households and individuals. Odds Ratios (OR) and their 95% confidence interval (95% CI) were calculated. Factors associated (p-value < 0.15) with recent dengue infection in univariate analysis were selected for multivariate analysis. In multivariate analysis we tested significant (p-value < 0.10) interactions between determinants and confounding. In the tables, variables for which the confidence interval does not include one are significant at the 0.05 level. Variables were mostly categorical, and continuous variables were categorized using quantiles.

Preliminary analysis of the data included several progressive steps. First, analyses of recent dengue infection on individual and household data were conducted survey by survey. Then, two-level multilevel analyses combining individual and household data were conducted per survey and site. The third step consisted of two-level analyses combining longitudinal and individual data, and longitudinal and household data. These analyses were useful in understanding seasonal and inter-annual dynamics and allowed confirmation of the final results. Here we only present the results of the three-level models, with survey, individual and household as the three different levels.

We linked the occurrence of recent dengue infection within a household with the landscape attributes of its surroundings. Based on land cover maps, landscape factors were calculated for each household. The legend of the land cover maps comprised:

1. Mixed deciduous forest.

2. Dry deciduous forest.

3. Bush or sparse forest.

4. Irrigated fields (wet): cultivated in March.

5. Irrigated fields (dry): not cultivated in March.

6. Old orchards (tree cover larger than 60%).

7. Water bodies and wide rivers.

8. Upland fields/young orchards (tree cover lower than 60%).

9. Sparsely vegetated area related to various human activities. No building or agriculture. (e.g. wasteland, grassy area)

10. Densely built areas.

11. Village zones with dense vegetation.

12. Village zones with sparse vegetation.

Variables related to land cover were: (i) the percentage of each land cover class in a 200-meter buffer (circle with a 200 meters radius) around each house; and (ii) the distance between each house and the nearest patch over 2,700 m^2 ^(i.e. at least 4 Landsat pixels) for each land cover class (except village zone classes). The distance from a house to the edge of the village was also calculated. Land cover was considered unchanged over the three years of the study, which was confirmed by field visits in each site and for each year of the epidemiological survey. Land-cover variables derived from the 2000 image can therefore be considered valid for the epidemiological survey years, from 2001 to 2003. Variables were categorized using quantiles. No other criterion such as mosquito flight-distance was used.

Furthermore, to investigate whether cases of recent dengue infections were clustered, analyses of clustering in time and in space were performed using the Kulldorff spatial scan statistic [24]. Clustering occurs when the probability of recent dengue infections is not randomly distributed. A circular moving window, with a continuously varying radius, was used to analyze village maps. Similarly, a temporal window was used to analyze the longitudinal data. For each window position, a statistic tested whether there was an increased risk of infection within as opposed to outside the window. The P-value was obtained from a likelihood ratio test based on Monte Carlo simulation with 9,999 replicates. The cluster analyses were performed per village using recent infections as cases and non-infections as controls. Houses were used as census areas. This method only detects circular clusters, which was assumed to be appropriate for the spread of a mosquito-borne disease, and it does account for varying house densities.

### Weather impact on dengue

The weather analysis was limited to deviations in temperature and rainfall of the survey years from the 1989–2003 average, for the following reasons. Weather data were only available for provincial capitals (Chiang Mai and Lamphun), the exact month of infection was unknown and weather variations are not constant between months within a season.

## Results

**Figure 2:**
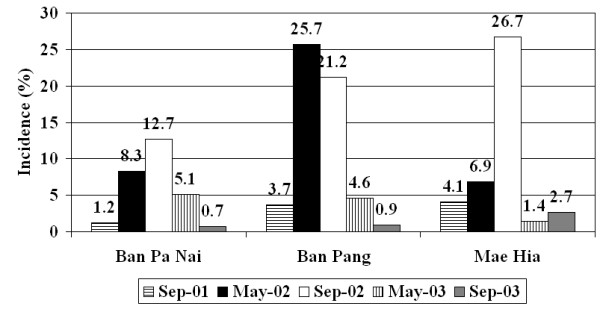
Incidence rate of recent dengue infection.

**Figure 3:**
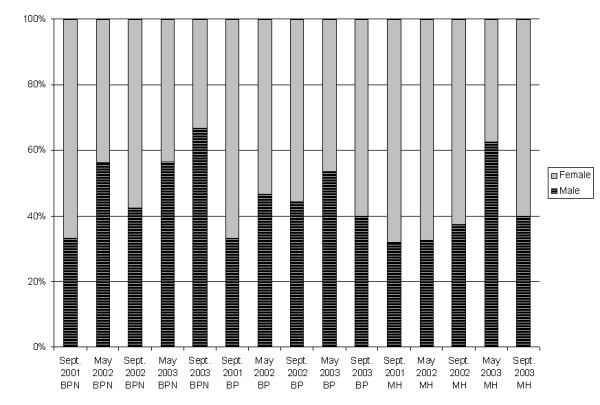
**Sex distribution of recently infected individuals**. BPN = Ban Pa Nai; BP = Ban Pang; MH = Mae Hia.

**Figure 4:**
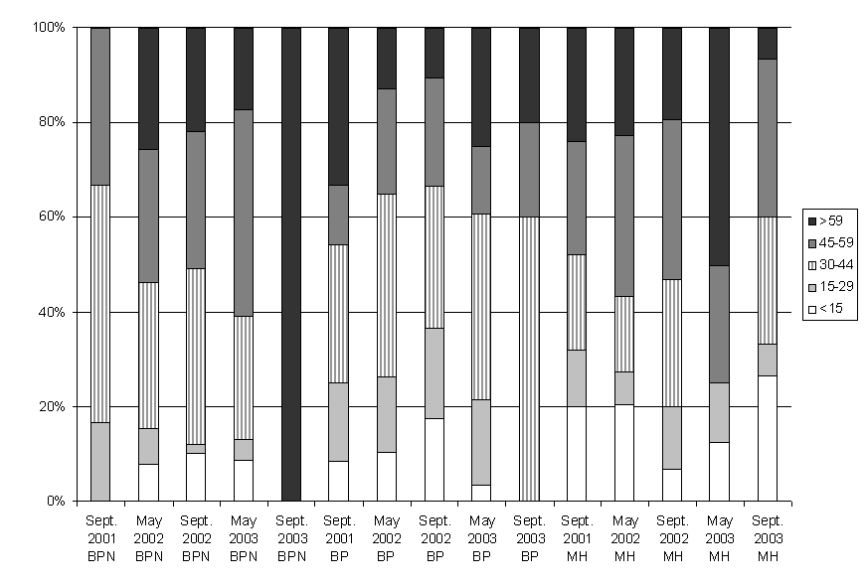
**Age distribution of recently infected individuals**. BPN = Ban Pa Nai; BP = Ban Pang; MH = Mae Hia.

Details of the study population have been presented before [13]. Briefly, in May 2001, 1928 persons were contacted and 1750 of them were followed-up in September 2001 (91%), constituting the individuals included in the study. Of these, 28% were in Ban Pa Nai, 37% in Ban Pang, and 35% in Mae Hia. Sex and age distribution differed per site (Table 1). Follow-up rates for the other surveys were 90%, 87%, 85% and 81%. Rates of recent infection varied over time and between sites (Figure 2, Table 2). Sex and age distribution of recently infected people also varied over time and between sites (Figures 3 and 4). Despite high rates of recent infection in several surveys, the number of cases reported in the calendar filled in daily was low: 6, 5 and 5 cases in 2001, 2002 and 2003, respectively, implying that most infections were asymptomatic. The data from the district-level public health surveillance system showed 1 case of dengue in Ban Pang in 2001, 1 case in Mae Hia in 2002 and 8 cases in Mae Hia in 2003. Therefore, the percentage of asymptomatic or lightly symptomatic dengue cases (public health systems records divided by recent infection data) varied over the years, ranging from 65% in 2003 to 99.7% in 2002. Although mostly relatively serious cases are reported to the public health authorities, the number of self-reported cases was also low, indicating that these rates are an appropriate estimate.

**Table 1:** Characteristics of the 1750 participants included in September 2001.

	Ban Pa Nai	Ban Pang	Mae Hia	Total	
	%	%	%	N	%
**Total**	28	37	35	1,750	100
					
**Sex**					
Male	47	48	40	786	45
Female	53	52	60	964	55
					
**Age (years)**					
< 15	14	15	13	249	14
15 – 29	11	19	15	268	15
30 – 44	28	31	24	483	28
44 – 59	25	21	26	420	24
> 59	21	14	22	330	19

**Table 2:** Number of recent dengue infections over the five surveys.

	Ban Pa Nai	Ban Pang	Mae Hia	Total
**September 2001**	6	24	25	55
**May 2002**	39	163	44	246
**September 2002**	59	131	160	350
**May 2003**	23	28	8	59
**September 2003**	3	5	15	23

Since determinants for recent dengue infection differed substantially between sites, results are presented separately by site. The intraclass correlations for household and individual levels were low, indicating that the lowest level of analysis, i.e. time period, explained most of the variance. There was a large variation in human behavior over time.

### Ban Pa Nai

Persons who mostly spent their daytime at school had a lower risk of recent dengue infection compared to other persons (Table 3). Bed nets protected against dengue infection as did houses made of a combination of wood, stone and concrete. Persons who went to the forest during daytime, spent their evening around the house or had dinner after 18.00 h had an increased risk of dengue infection. People living within 60 m of irrigated fields had a higher risk of infection. In contradiction with this, households surrounded for more than 40% by irrigated fields had a two times lower risk.

**Table 3:** Three-level analyses to identify determinants for recent dengue infection in Ban Pa Nai, northern Thailand.

	% sc^a^	aOR^a^	95% CI^a^
**ICC individuals**		<0.0001	
**ICC households**		0.058	
			
**Time (survey)**			
May 2002	8.3	1.0	
September 2002	12.7	1.60	1.01–2.55
May 2003	5.1	0.63	0.36–1.10
September 2003	0.7	0.08	0.02–0.27
*Individual-level variables*:			
**Spend days**			
School	2.5	1.0	
House	6.2	3.06	1.16–8.08
Fields	6.1	3.22	1.20–8.66
Forest	4.8	1.97	0.19–20.5
Factory/office	7.9	3.05	0.74–12.5
Other	5.1	2.58	0.69–9.65
**Eat time**			
< 18.00 h	4.4	1.0	
>= 18.00 h	6.6	1.70	1.09–2.65
**Using bednets**			
No	8.7	1.0	
Yes	5.3	0.43	0.24–0.80
**Days in forest**			
No	6.2	1.0	
Yes	12.3	1.75	0.99–3.07
**Evening around house**			
No	5.6	1.0	
Yes	8.2	1.52	1.01–2.29
*Household-level variables*:			
**Housing**			
Wood or bamboo	5.9	1.0	
Stone	7.8	1.10	0.61–2.00
Combination of stone, wood/bamboo	4.1	0.59	0.36–0.96
**Distance to irrigated fields**			
0–60 m	7.6	1.0	
>60 m	4.3	0.45	0.29–0.70
**% of irrigated fields in 200 m**			
0–40%	6.5	1.0	
>40%	4.8	0.51	0.33–0.78

^a ^sc = recent dengue infection; aOR = adjusted Odds ratio; 95%-CI = 95% Confidence Interval.

### Ban Pang

Students, unemployed persons and laborers had a lower risk of dengue infection compared to housewives, farmers and traders (Table 4). Spending daytime inside the house or in the fields decreased the risk, as did spending the evening inside the house. No important preventive measures were identified. Persons who lived in houses not surrounded by water containers or without domestic animals had a lower risk. In contrast to Ban Pa Nai, persons who lived further away from irrigated fields (> 600 m) had a higher risk. In contradiction with this, persons in households with irrigated fields present within 200 m had a higher risk than persons living in households with no irrigated fields in the surrounding.

**Table 4:** Three-level analyses to identify determinants for recent dengue infection in Ban Pang, northern Thailand.

	% sc^a^	aOR^a^	95% CI^a^
**ICC individuals**		<0.0001	
**ICC households**		<0.0001	
			
**Time (survey)**			
May 2002	25.7	1.0	
September 2002	21.2	0.84	0.64–1.11
May 2003	4.6	0.14	0.09–0.21
September 2003	0.9	0.03	0.01–0.07
*Individual-level variables*:			
**Profession**			
Farmer	12.6	1.0	
Trader	15.1	1.08	0.56–2.09
Housewife	14.7	1.15	0.69–1.90
Student	9.0	0.51	0.34–0.77
Other	12.8	0.55	0.26–1.16
Unemployed	8.3	0.55	0.30–0.99
Labour	10.0	0.63	0.43–0.91
**Days in house**			
No	19.8	1.0	
Yes	10.5	0.71	0.54–0.94
**Days in fields**			
No	14.1	1.0	
Yes	12.5	0.66	0.48–0.90
**Evening in house**			
No	23.4	1.0	
Yes	12.6	0.66	0.43–1.02
*Household-level variables*:			
**Water around house**			
Yes	12.2	1.0	
No	9.4	0.63	0.46–0.86
**Domestic animals**			
Yes	12.3	1.0	
No	9.8	0.79	0.60–1.04
**Distance to irrigated fields**			
0–600 m	9.4	1.0	
601–800 m	13.9	1.88	1.32–2.67
>800 m	11.1	1.50	1.01–2.21
**% irrigated field in 200 m**			
0%	11.7	1.0	
>0%	10.4	1.38	0.95–2.00

^a ^sc = recent dengue infection; aOR = adjusted Odds ratio; 95%-CI = 95% Confidence Interval.

### Mae Hia

People who used abate (a larvicide targeting all mosquito species and other insects) had a higher risk of dengue infection (Table 5). People who reported to be sometimes or often bitten by mosquitoes had a higher risk than people who reported to be never bitten. Persons living in houses made of wood had a lower risk compared to persons living in other houses. People living further away (> 300 m) from orchards had a lower risk compared to persons living within 150 m from orchards. Persons living more than 500 m from a lake or river or in a part of the village with little vegetation had a higher risk of infection. Persons having bare soils in the surrounding of their house had a lower risk.

**Table 5:** Three-level analyses to identify determinants for recent dengue infection in Mae Hia, northern Thailand.

	% sc^a^	aOR^a^	95% CI^a^
**ICC individuals**		<0.0001	
**ICC households**		<0.0001	
			
**Time (survey)**			
May 2002	6.9	1.0	
September 2002	26.7	4.76	3.28–6.91
May 2003	1.4	0.18	0.08–0.39
September 2003	2.7	0.31	0.16–0.57
*Individual-level variables*:			
**Using abate**			
No	5.8	1.0	
Yes	9.7	1.42	0.99–2.02
**Bitten during day time**			
Never	7.2	1.0	
Sometimes	8.9	1.25	0.88–1.78
Often	8.6	1.71	0.90–3.26
*Household-level variables*:			
**Housing**			
Wood or bamboo	7.6	1.0	
Stone	7.8	1.27	0.79–2.04
Combination of stone, wood/bamboo	10.3	1.90	1.16–3.10
**Distance to orchards**			
0–150 m	9.6	1.0	
151–300 m	8.8	0.72	0.49–1.04
>300 m	7.1	0.47	0.28–0.79
**Distance to water bodies**			
0–500 m	5.9	1.0	
500–1300 m	8.3	2.26	1.32–3.87
>1300 m	10.5	2.66	1.49–4.75
**% bare soils in 200 m**			
0%	9.8	1.0	
1%	7.8	0.74	0.49–1.11
>1%	7.3	0.55	0.38–0.79
**% village area with vegetation in 200 m**			
0–2%	8.4	1.0	
3–8%	8.3	0.73	0.49–1.10
>8%	8.7	0.54	0.68–0.79

^a ^sc = recent dengue infection; aOR = adjusted Odds ratio; 95%-CI = 95% Confidence Interval.

The results of the three-level models were consistent with the results of the one and two-level models (results not shown).

### Cluster analyses

**Figure 5:**
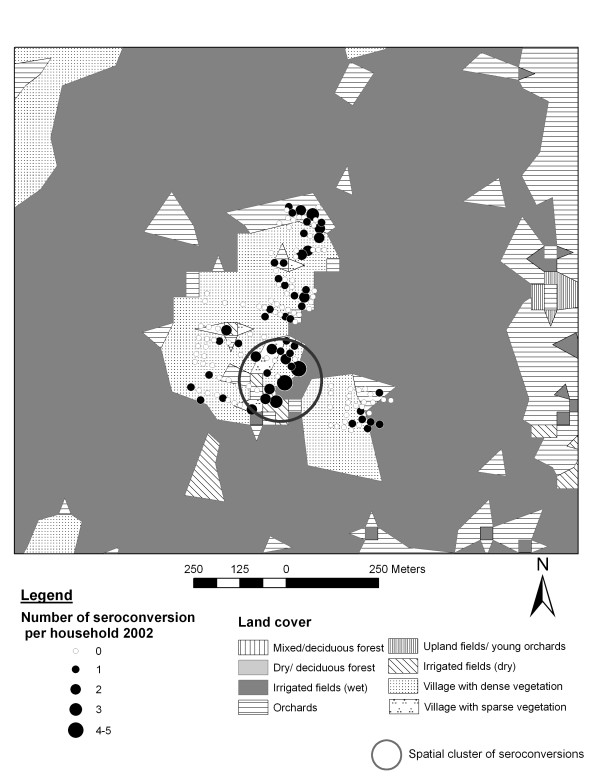
Land cover, recent dengue infection in 2002 and spatial cluster in Ban Pa Nai.

**Figure 6:**
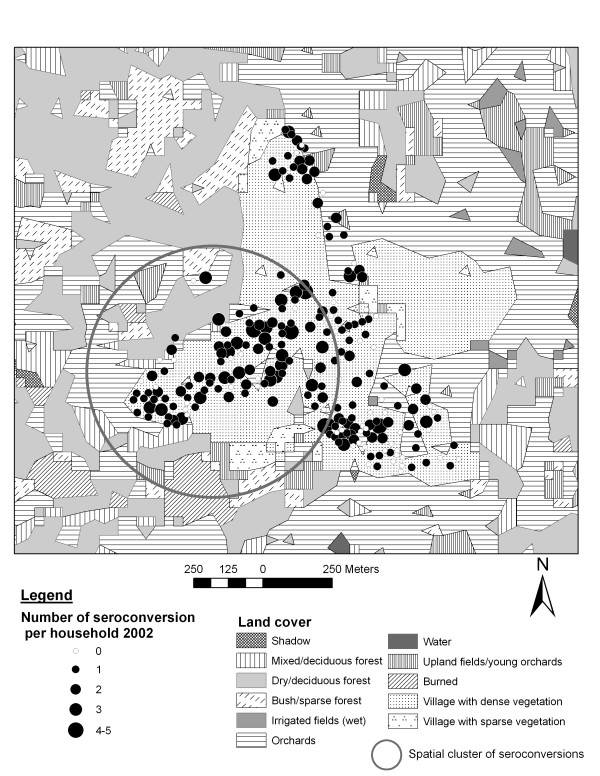
Land cover, recent dengue infection in 2002 and spatial cluster in Ban Pang.

**Figure 7:**
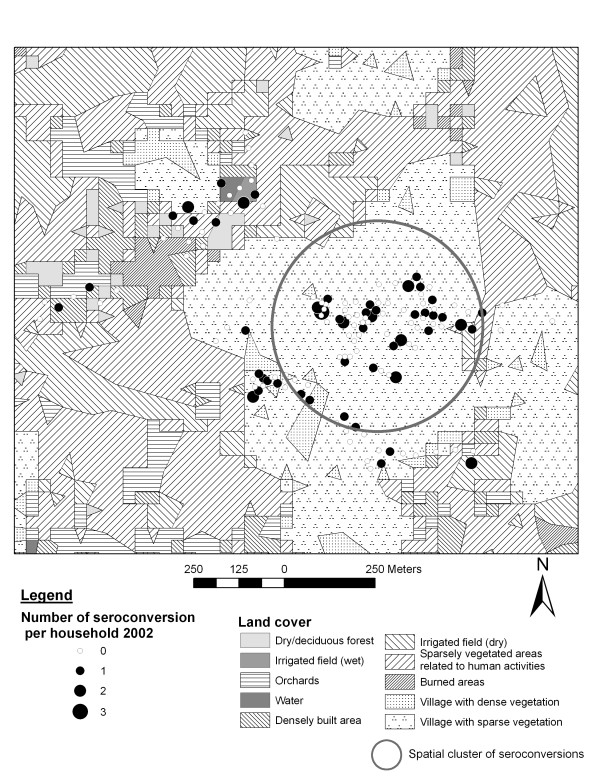
Land cover, recent dengue infection in 2002 and spatial cluster in Mae Hia.

In all three sites the number of recently infected people was significantly higher in 2002 than in 2001 and 2003 (p < 0.0001), indicating temporal clustering of recent dengue infection. In 2002, there were 72 observed instead of 38.2 expected cases in a spatial cluster in Ban Pa Nai, 274 observed instead of 131.2 expected cases in Ban Pang, and 192 observed against 66.2 expected cases in Mae Hia. Within these temporal clusters, significant spatial clusters were identified: two in Ban Pa Nai, one in Ban Pang and one in Mae Hia (Figure 7). In Ban Pa Nai (Figure 5; only one village and one cluster shown), the most significant cluster was located in the southern part of the village, near the edge of the village, and therefore near irrigated fields, but in this case also near another part of the village. In Ban Pang, the cluster was located in the western end of the village, far from the irrigated fields (Figure 6). In Ban Pa Nai, one other spatial cluster was found in September 2003; 15 cases were observed whereas 4.7 were expected (p = 0.004).

### Weather

The weather pattern in Chiang Mai and the infection rate were not strongly correlated. In 2001 the temperature of nine months differed significantly from the 1989–2003 average temperature in these months, including six colder months, whereas in 2002 and 2003 this figure was three months (t-test, p < 0.05), including two colder months each year. For rainfall, 2001 had six months significantly different from the 1989–2003 average rainfall for these months (three months dryer than the average). 2002 had eight anomalous months (three months dryer) and 2003 had seven (four months dryer). 2002 was thus more different in terms of rainfall than in terms of temperature. The largest variations in rainfall are observed in the wet season (May to October). 2002 had extreme rainfall in November and December.

## Discussion

To study the personal, household and environmental determinants of recent dengue infection, and its pattern in space and time, we conducted a prospective cohort study in three dengue-endemic sites in Chiang Mai and Lamphun provinces, Thailand. By following a population biannually during three consecutive years, we located recently infected individuals in space and time by analyzing dengue-specific antibody levels during each survey.

### Individual-level risk determinants

Factors varying over time, that explain the largest part of the variance, included several individual level determinants that were related to the location where people spent their daytime and evenings. In both rural sites, students at school had the lowest relative risk. This possibly relates to the existing intensive prevention programs in and around schools, involving for example breeding site elimination. Being outside the house during daytime or evening increased the risk of dengue infection suggesting that transmission takes place outside the house in rural areas, whereas this was not the case in the peri-urban study site. Eating after 18.00 h can be associated with activities outside the house taking place later. The identification of clusters of cases in neighboring houses suggests that transmission also takes place in or around the house. The decrease in risk associated with days spent in fields is associated with the absence of breeding sites in field cropping areas, whereas days spent in the house, near sources of mosquitoes, increased the risk. The increase in risk associated with the time spent in the forest is not well understood, since *Ae. aegypti *and *Ae. albopictus *were not found in the forest in the study area [Vanwambeke et al., forthcoming].

Several studies showed that dengue risk exposure is more important in and around the house because female *Ae. aegypti *are highly domesticated, and *Aedes *mosquitoes mostly bite during daytime with pronounced peaks of activity around sunrise and sunset [25,26]. Activity can be prolonged at night in urbanized areas, possibly due to the higher light intensity at night [26]. Field observations in our study area, however, suggest that *Ae. albopictus *is found in villages and in orchards, [Vanwambeke et al., forthcoming], where people could also be infected during peak biting times. Several other studies suggest that *Ae. albopictus *probably serves as a maintenance vector of dengue in rural areas of South-East Asia [27,28]. In terms of infection control, this indicates that larval control around houses is relevant, but also that prevention of bites in or around the house could make substantial contribution to the control of infection, as well as similar measures in orchards.

In Mae Hia, being bitten during the day increased the risk of dengue infection, which directly relates to the vector activity. It is however not clear whether this determinant relates to the mosquito population or to the exposure to biting. Also, this variable describes subjective reporting by people, with no distinction between *Aedes *bites and bites received from other genera or even taxa.

### Preventive measures

In Thailand, dengue control is focused on vector elimination rather than personal protection. The use of abate (a larvicide) in Mae Hia and the use of bed nets in Ban Pa Nai were the only preventive measures related to dengue infection in our study. The use of abate in Mae Hia was actually related to an increase in the risk of dengue infection, suggesting that this preventive measure was applied too late when the larval population had already reached high levels, or was applied incorrectly, for example by not treating all containers. In two villages in North-Eastern Thailand, Eamchan *et al*. [29] observed only limited success with the use of abate: not all containers were treated or covered. Also, the relationship between the use of preventive measures and disease prevalence is not always straightforward, as was observed by Thomson *et al*. [30]. The use of bednets in The Gambia was highly correlated to the density of mosquito, whereas the disease prevalence could not easily be related to bednet use. Rosenberg *et al*. [31] also raised the possibility that, in a village in southeastern Thailand, bednets were used mostly when the risk of infection was low but the nuisance of mosquitoes highest. Information on the level of nuisance caused by mosquitoes or on the observable level of the larvae population, and on the way in which people apply abate in and around their house would help in understanding these results.

The protective effect of bednets in Ban Pa Nai is unexpected when accounting for the fact that *Aedes *mosquitoes do not bite at night-time. However, it could be related to the early-morning peak of biting activity of *Aedes *mosquitoes when many people are still in bed. It could also protect children during the day. The potential role of bednets in preventing dengue infection was mentioned by Thavara *et al*. [32]. It is worth remembering also that many previous studies were focused on urban areas, whereas here the significant preventive effect of bednets was observed in a rural setting. The relation between the use of bednets and other individual or household-level characteristics such as knowledge of dengue was tested but no association was found. Again, more locally-collected information about the timing of activity peaks in mosquitoes, in relation to people's activity timing, would help to interpret the protective effect of bednets. The impact of the use of electric light at night could influence activity times for *Aedes *mosquitoes [25].

Generally, few preventive measures had statistically significant effects. Their use had even contradictory effects as shown by the case of abate larvicide. This suggests that the timing of prevention is crucial. The use of bednets could have a more important role in dengue infection prevention than previously thought, as indicated by its significant protective effect.

Household level effects included the type of housing in Ban Pa Nai and Mae Hia. People living in houses made of a combination of materials had a lower risk; the causal link behind this variable is not clear. In Ban Pang the significant household variables are related to the vector ecology. Houses with no water containers around the house, therefore providing no breeding sites, had a lower risk. Houses with no domestic animals had a lower risk as well. Animals could provide alternative blood sources. However, *Aedes *mosquitoes are highly anthropophilic. The presence of animals might enhance the attractivity of the house, but mosquitoes would only bite humans.

### Landscape and land-cover variables

Land cover may be an important risk determinant for infection, depending on whether the landscape surrounding a person supports a large mosquito population or not, mostly by providing breeding habitats. In this study, we attempted to directly relate landscape features with the risk of dengue infection, by-passing a quantification of mosquito population in different habitats. The results indicate that land cover and spatial organization of villages and surrounding landscape play a role in dengue infection. However, great care in interpreting results related to land cover is needed. In Mae Hia, results highlight the role of land cover as a source of breeding habitat. Orchards often contain a variety of artificial water containers, which would offer alternative breeding sites for *Aedes *while improved housing conditions offer less breeding habitats around houses. Proximity to orchards increased the risk of infection in Mae Hia, whereas the presence of bare soils around the house decreased that risk. Bare soils are unsuitable for *Aedes *breeding. Other variables were not easy to interpret, such as the distance to water bodies. This could be a proxy for other features, including socio-economic variables. The variable related to the importance of village area with dense vegetation does not correspond to breeding preferences of *Aedes *mosquitoes but could be related to housing type or quality.

Location of possible clusters and landscape spatial pattern should be considered together when interpreting the land cover effects on infection risk. In Ban Pa Nai, the apparent contradiction of the effect of proximity to irrigated field decreasing the risk on the one hand and of the proportion of irrigated field effect (decreasing the risk with increasing proportion) on the other hand is caused by the particular spatial configuration of the villages and the location of the cluster. The cluster is indeed located near the edge of the village, but with few irrigated fields around. Irrigated fields offer no suitable breeding habitat for *Aedes *and therefore do not act as a source of mosquitoes, whereas village areas do.

A similar effect was observed in Ban Pang: the location of the cluster further away from the irrigated fields explains the effect of distance to irrigated fields (wet), while a higher risk for people living with a higher proportion of irrigated fields around possibly proxies another effect. This variable is significantly correlated with none of the other landscape characteristics. The orchards found in this area are much older and possibly influence mosquito breeding and transmission differently than younger orchards. These houses are also located close to the main road.

Our results indicate that land cover needs to be considered in dengue transmission dynamics, especially land-cover types providing breeding habitats, but that varying local conditions will strongly influence the importance and role of the landscape on the risk of infection. Previously unused habitats, such as orchards, might be found in increasingly important land-cover types in a context of housing improvement as observed in the suburbs of Chiang Mai. Agricultural land covers can no longer be ignored in dengue control given the rising prevalence of dengue in rural areas. Improved knowledge on vector ecology, behavior and dispersal, especially regarding *Ae. albopictus*, and on the role of this vector in dengue transmission would greatly improve interpretation of land cover effects [Vanwambeke et al., forthcoming]. GIS can be a useful tool since it integrates spatial and environmental variables and locates cases of infection to identify high-risk areas and environmental determinants. The spatial configuration of villages needs to be considered when considering spatial patterns of infection and a thorough knowledge of vector ecology can also help in understanding the observed patterns.

### Cluster analyses

The peak incidence of recent dengue infection took place in 2002 for all study sites, corresponding with the temporal cluster identified by analysis of spatial and temporal clustering. During this peak year, some areas within a site were more affected than others, indicating variation in local infection patterns. As was shown in the multi-level analysis, the determinants for recent dengue infection differed between sites and, as shown by the spatial clusters, possibly within sites. Moreover, the intraclass correlation for individual and household level was very low, thus the largest part of variance was explained by factors varying over time: conditions might have been more favorable in 2002. These results show that the focal nature of the infection does not only exist for symptomatic dengue cases but also occurs in asymptomatic infections, and indicates the relevance of studying asymptomatic dengue infection.

The clusters could also relate to host-pathogen dynamics. A radiating pattern emanating from Bangkok has been described by others [5,33,34]. The radiating pattern in symptomatic dengue cases is thought to reflect host-pathogen population dynamics [35], but what causes the wave pattern of asymptomatic infections is unknown. It might be related to changes in the year-round circulation of dengue virus [36,37], since infection took place both between May and September and between September and May. Serotypes were not measured in this study, and the peak of infections in 2002 observed in our study did not correspond with the peak in symptomatic dengue cases that occurred in 2001. National data showed that the main serotype in 2000 and 2001 was serotype 1, whereas in 2002 serotype 2 was the main serotype detected (Ministry of Public Health, Dept. of Disease Control). The percentage of asymptomatic or lightly symptomatic infections was high but varied over the years as was shown by others [10,14]. The focal nature of dengue in space and time was observed in other studies and could be related to clusters of *Aedes *[34,38,39].

### Limitations of the study

Factors determining the spatial and temporal clustering were not specifically investigated in this study, but would deserve further work. Clustering could have several origins: host-pathogen dynamics, national scale radiating patterns of cases, mosquito-vector ecology, or individual or household-level risk determinants. Such a study would however require a different data collection approach.

Note also that no household-level entomological data were collected as part of this study.

## Conclusion

Our study showed that the focal nature of dengue not only appears in symptomatic dengue cases, but also exists for asymptomatic dengue infections. Environmental variables were significant, but the interpretation of their effect needs to combine several types of information such as the existence and location of clusters and vector ecology. The great variation of determinants for recent dengue infection in space and time should be taken into account when designing local dengue control programs.

## Authors' contributions

Sophie O. Vanwambeke derived land-cover maps from the Landsat image and derived land-cover variables for the surveyed households. She participated in analyzing the dengue infection data. She analyzed weather patterns in the survey years as presented. She led the writing of the paper. Birgit H.B. van Benthem directed the epidemiological data collection, and digitized the village maps. She participated in analyzing the dengue infection data. She carried out the three-level analysis including individuals, households and all surveys as presented, and the cluster analysis on the longitudinal data. SOV and BvB interpreted the results together and then with the other co-authors. Nardalada Khantikul organized, supervised and participated in the collection of the dengue data. Chantal Burghoorn-Maas carried out the laboratory analysis of blood samples. Kamolwan Panart supervised and participated in the collection of the dengue data. Linda Oskam participated in conceptualizing the collection of the dengue data and analysis, in interpreting results, and provided input on the paper. Eric F. Lambin participated in conceptualizing the land-cover data analysis, in interpreting results, and provided input on the paper. Pradya Somboon helped designing and supervising the collection of the dengue data, in interpreting results, and provided input on the paper.

## Financial support

This study was financially supported by EU grant QLRT-1999-31787, provided within the Quality of Life and Management of Living Resources Programme (1998–2002).

No conflict of interest to declare.
